# Feasibility and Safety of the Distal Transradial Artery for Coronary Diagnostic or Interventional Catheterization

**DOI:** 10.1155/2020/4794838

**Published:** 2020-12-09

**Authors:** Yaowang Lin, Xin Sun, Ruimian Chen, Huadong Liu, Xinli Pang, Jie Chen, Shaohong Dong

**Affiliations:** ^1^Department of Cardiology, Shenzhen Cardiovascular Minimally Invasive Medical Engineering Technology Research and Development Center, Shenzhen People's Hospital, The Second Clinical Medical College, Jinan University, Guangzhou, China; ^2^The First Affiliated Hospital, Southern University of Science and Technology, Shenzhen 518020, Guangdong, China

## Abstract

**Background:**

This prospective study compared the success rate and safety of a distal transradial artery (dTRA) approach to that of the conventional transradial artery (TRA) for coronary angiography or percutaneous coronary intervention.

**Methods:**

From January 2019 to April 2020, nine hundred consecutive patients (height < 190 cm) scheduled for coronary angiography or percutaneous coronary interventions were randomly and equally assigned to receive either dTRA or conventional TRA catheterization.

**Results:**

Successful access was achieved in 96.00% and 96.67% of the dTRA and conventional TRA groups, respectively (*P*=0.814). Compared with the TRA group, patients in the dTRA experienced significantly less hemostatic band removal time (150.5 ± 50.5 cf. 210.6 ± 60.5 min, *P*=0.032); minor bleeding of the access site (2.44% cf. 6.44%, *P*=0.038); hemostatic band cost (USD; 0.1 cf. 59.4, *P*=0); and postprocedural radial artery occlusion (1.56% cf. 3.78%, *P*=0.035). A lower body mass index was a higher risk factor for dTRA access failure (odds ratio = 0.79, *P*=0.024), with a cutoff of 22.04 kg/m^2^.

**Conclusion:**

Compared to conventional TRA, dTRA had a comparable high success rate, with fewer associated complications. Clinicians should use the dTRA with caution in patients with low body mass index.

## 1. Introduction

For decades, most coronary interventions were performed via the femoral approach. Currently, radial artery catheterization is used for more than 90% of catheterization laboratory procedures because of fewer access-related complications [1–3], better patient comfort [4], and faster mobilization [5].

Catheterization by transradial artery (TRA) has advantages, but also disadvantages that include minor bleeding at the access site and radial artery occlusion after the procedure. Accordingly, the distal transradial artery (dTRA) approach in the anatomical snuffbox may be an alternative site for radial artery puncture because of lower radiation exposure, less radial artery damage, and shorter radial compression time [6]. However, few prospective studies have evaluated the feasibility of dTRA with predictors of access failure.

This study evaluated the rate of success, postprocedural complications, and predictors of cannulation failure of the dTRA relative to the conventional TRA approach.

## 2. Methods

### 2.1. Study Design and Patients

This was a single-center prospective clinical study. From January 2019 to April 2020, 900 consecutive patients who underwent coronary angiography or percutaneous coronary intervention were prospectively included. Patients with any of the following were excluded: height >190 cm; without obvious pulsation of the radial artery; cardiogenic shock; or aged >80 years.

The enrolled patients were separated into dTRA group (*n* = 450) or conventional TRA group (*n* = 450) by envelope randomization at the Department of Cardiology, Shenzhen People's Hospital. The institutional review board at Shenzhen People's Hospital approved the study protocol.

### 2.2. Radial Artery Cannulation Procedure

**Figure 1:**
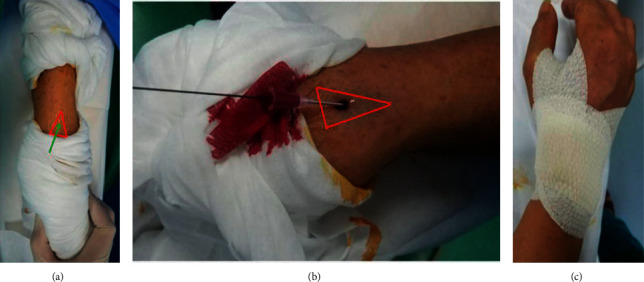
Catheterization by dTRA on the right side. (a) Patient's position for the right snuffbox approach. The arm is pronated with the anatomical snuffbox upward. (b) Insertion of the 6 French introducer sheath (Terumo). (c) Hemostasis of the puncture site by manual compressive bandage with gauze.

All catheterization procedures were performed by either of 2 doctors who were experienced in dTRA and TRA on the right hand. If radial artery cannulation failed, the femoral access approach was an alternative. In the dTRA group, the arm was pronated with the anatomical snuffbox kept upward (Figure 1(a)). Before the puncture, the patient's blood pressure was recorded as well as heart rate. All the punctures were done using an intravenous catheter needle (1.02 mm) with 6 French introducer sheath (Radifocus introducer II, Terumo Medical; Figure 1(b)). Subsequent to successful cannulation, unfractionated heparin (50 IU/kg) combined with nitroglycerin (0.5 mg) was administered intrasheath. After the procedure of catheterization, a hemostatic band (Kangdelai Medical Devices, Shanghai China) was used for hemostasis in the TRA group, while manual compressive bandage with gauze (Figure 1(c)) was applied for covering the puncture site in the dTRA group. In the TRA group, the band was slowly compressed through a band-tightening press until bleeding was stopped, and the time of band application was recorded. After compression for 120 minutes, the band setscrew was loosened 1 lap every 15 minutes. If bleeding occurred, the band was rescrewed with the same lap and was observed for 30 minutes. Finally, the hemostatic band removal time was registered. In the dTRA group, the manual compressive bandage with gauze started to loosen after compression for 120 minutes. Bleeding was observed every 15 minutes until the bandage was removed. Before discharge, the patency of the radial artery at the site of puncture was evaluated using the Doppler ultrasound (Handydop Pro, Medisound Medical Apparatus Co. Ltd.) evaluation.

### 2.3. Study Endpoints

The primary endpoint was the success rates of the catheterizations, with success defined as a successfully cannulated sheath. Secondary endpoints were complications of the two groups and possible predictors of cannulation failure in the dTRA group.

### 2.4. Statistical Analysis

The data analysis was carried out with IBM SPSS 22.0 software. Values in the tables are shown as mean ± standard deviation or count (percentage). Student's *t*-test or the Kruskal–Wallis test was employed for between-group differences of continuous variables. The chi-squared test or Fisher's exact test was used for categorical variables. Multivariate logistic analysis was conducted to determine possible predictors of cannulation failure in patients given the dTRA. A receiver operating characteristic curve analysis was performed to calculate the area under the receiver operating characteristic (AUC) curve, cutoff point, sensitivity, and specificity of risk factors of failure in the dTRA group. Statistical significance was considered *P* < 0.05.

## 3. Results

### 3.1. Baseline Characteristics

The mean ages of the dTRA and TRA groups were 55.28 ± 10.59 and 58.81 ± 9.42 years, respectively (*P*=0.075; Table 1). The groups were statistically similar with regard to the following: demographic characteristics; body mass index (BMI); systolic blood pressure (SBP) and diastolic blood pressure (DBP) at the cardiac catheterization (cath) lab; risk factors of coronary artery disease; previous cardiac catheterization; left ventricular ejection fraction (LVEF); diagnostic catheterization or interventional catheterization; and medical treatment.

### 3.2. Success Access Rate and Complications in the dTRA and TRA Group

**Figure 2:**
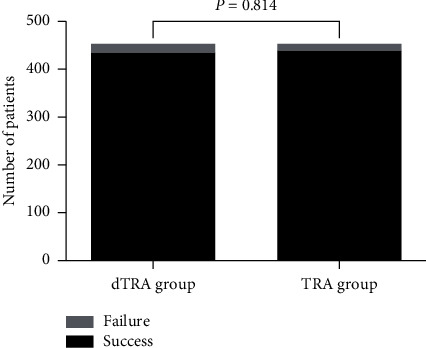
Success and failure of radial artery cannulation in the dTRA and TRA groups. The success rates were 96% (432/450) and 96.67% (435/450) in the dTRA and TRA groups, respectively (*P* = 0.814).

The success rates of cannulation of the dTRA (432/450 or 96.0%) and conventional TRA (435/450 or 96.67%) groups were comparable (*P*=0.814; Figure 2). Ten patients were converted to TRA and 8 patients to the femoral approach in the failed dTRA group, while 6 patients were successfully converted to the dTRA approach and 9 patients converted to the femoral approach in the failed TRA group.

The success rates, access times, and rates of hematoma were similar between the dTRA and TRA groups (Table 2). However, the following were significantly lower in the dTRA compared with the TRA: hemostatic band removal time; minor bleeding of the access site; cost of the hemostatic band; and postprocedural radial artery occlusion.

### 3.3. Predictors of Cannulation Failure in Patients with dTRA

**Figure 3:**
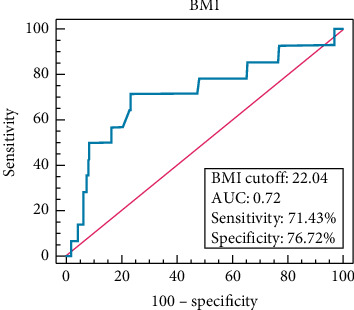
The AUC of BMI for failure in the dTRA group. The AUC was 0.72 (95%CI, 0.668 to 0.769). The cut-off baseline value for BMI was set to 22.04 with specificity of 76.72% and sensitivity of 71.43%, respectively.

**Figure 4:**
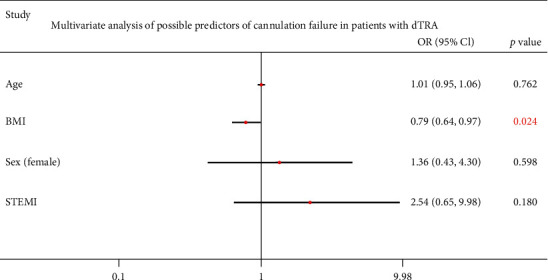
Multivariate analysis of possible predictors of cannulation failure in patients with dTRA.

Potential factors that might favor failed cannulation via dTRA were subjected to logistic multivariable analysis (Table 3). Only low BMI was a risk factor of cannulation failure (OR = 0.79, *P*=0.024) with a cutoff value of 22.04 kg/m^2^ (specificity of 76.72%, sensitivity of 71.43%, AUC 0.72; Figure 3). Only 48 (10.67%) patients with ST-segment elevation myocardial infarction (STEMI) were included, with a successful dTRA access rate of 90.91%. The present study did not find that STEMI was a significant risk factor for failure of dTRA cannulation (OR = 2.54, *P*=0.180; Figure 4), although it may be logical to assume that it might be.

## 4. Discussion

This prospective clinical research determined that dTRA is a feasible choice for either selective or emergency catheterizations. It is the first clinical trial to analyze possible predictors of cannulation failure in patients with dTRA. It was found that dTRA may be not the best option for the patient with lower BMI.

The anatomical snuffbox is at the dorsal aspect of the wrist, with a triangular shape (Figure 1(a)) [7]. The distal radial artery is derived from the radial artery and is located at this narrow snuffbox. Babunashvilli and Dundua [8] initially used the dTRA for dilating occluded radial arteries, while Kiemeneij [6] was the first to introduce the left dTRA as an access site for coronary intervention treatment. According to a review of the literature, the rate of successful cannulation using dTRA is 76.3% to 100% [9–14]. In the present study, the dTRA in the snuffbox area was successful in 96.00% of attempts, which is in accordance with Lee et al. [11] and Roghani-Dehkordi et al. [12]. However, in the only other published randomized trial regarding dTRA vs. TRA cannulation for coronary angiography, the success rate of dTRA cannulation was only 79–89% [15]. The possible reasons for this difference were (1) doctors in our study were more skilled in dTRA punctures with more than 100 procedures each, (2) only 10.67% STEMI patients were included, which was a potential risk factor for failure of dTRA cannulation (OR = 2.54, *P*=0.180) with a successful dTRA access rate of 90.91%, (3) the number of attempts and skin punctures were not restricted in the dTRA group (a higher mean puncture attempts), (4) wire improvement with a 0.025-inch wire head end shaped with a little bend, or a 0.021-inch plastic wire.

The dTRA has numerous advantages over TRA. First, the dTRA maintains the antegrade flow with no damage to the superficial palmar arch bifurcation, meaning that the risk of radial artery occlusion is lower compared with TRA. Secondly, from the present clinical experience, the hemostasis time of dTRA is shorter than that of TRA (150.5 ± 50.5 cf. 210.6 ± 60.5 min, *P*=0.032), which contributes to the greater comfort of the patient. Moreover, postprocedural minor bleeding at the access site was significantly less with dTRA (2.44% cf. 6.44%, *P*=0.038) because of the easy hemostasis; the access artery has a smaller diameter and is surrounded with hard structures in the distal RA in the snuffbox. Third, dTRA saves the conventional radial approach in reserve for further procedures, especially for patients with potential multivessel disease who need coronary interventions repeatedly or patients with chronic kidney disease who require formation of an arteriovenous fistula for hemodialysis. The snuffbox approach spares the site for future repeated percutaneous coronary intervention or arteriovenous fistula, avoiding puncturing the femoral artery [16]. Finally, the dTRA serves as an approach for reverse interventional treatment in a setting of occluded radial arteries subsequent to TRA.

Although, in the present study, the dTRA had a demonstrated high success access rate, it is not always feasible, especially for the patient with a lower BMI. From our multivariate analysis, patients with BMI <22.04 were not optimal for dTRA. This may be because the distal radial artery in the anatomical snuffbox of patients with lower BMI is of small caliber and tortuous shape and therefore more difficult to puncture successfully. Soydan and Akin [17] reported that they successfully inserted the cannula via the left snuffbox with a 0.018-inch stainless steel wire in all 54 of their study patients. In our experience, a 0.025-inch wire head end shaped with a little bend or a 0.021-inch plastic wire (Glidesheath Slender, Hydrophilic Coated Introducer Sheath, Terumo Medical) is an alternative when there is difficulty in wiring after successful puncture. Finally, the dTRA may not be feasible in patients with lower BMI because of more anatomic variations underneath the snuffbox of these patients, in addition to the tortuosity and thinness of the radial artery.

Limitations should be acknowledged in the present study. First, only 48 (10.67%) patients with ST-segment elevation myocardial infarction (STEMI) were included. More relevant data are required to provide more convincing evidence. Secondly, it is difficult for tubes or catheters to reach the coronary ostium from the dTRA, especially in situations involving a tortuous axillary artery or a tall man. Accordingly, at our center, the dTRA approach is not considered an option for patients taller than 190 cm. This might have influenced the results related to BMI. Thirdly, the pain grade at access site was not recorded and vascular ultrasonogram was not used, which may have added more confirmation regarding the feasibility of the dTRA approach. Finally, this study is from a single center, and all procedures were performed by only 2 experienced radial operators. However, these results warrant larger randomized controlled trials for confirmation.

## 5. Conclusion

In conclusion, this study highlighted the feasibility and advantages of the dTRA approach for coronary angiography or percutaneous coronary intervention. The dTRA may not be the optimal approach for patients with low BMI.

## Figures and Tables

**Table 1:** Baseline characteristics of the dTRA and TRA groups ^*∗*^.

		dTRA	TRA	*P*
Subjects, *n*		450	450	
Age, years		55.28 ± 10.59	58.81 ± 9.42	0.075
Males		205 (45.56)	225 (50.00)	0.386
Body mass index, kg/m^2^		24.06 ± 3.58	24.36 ± 2.64	0.230
SBP at the cath lab, mmHg		129.15 ± 13.78	127.01 ± 14.61	0.190
DBP at the cath lab, mmHg		74.51 ± 11.08	75.46 ± 10.98	0.091
Heart rate at the cath lab, bpm		76.29 ± 12.09	76.89 ± 11.56	0.810
Hypertension		112 (24.89)	113 (25.11)	0.856
Diabetes mellitus		48 (10.67)	56 (12.44)	0.166
Current smoking		124 (27.56)	101 (22.44)	0.144
Previous cardiac catheterization		32 (7.11)	33 (7.33)	0.980
eGFR		80.45 ± 22.61	77.3 ± 26.53	0.528
LVEF, %		56.5 ± 12.6	57.3 ± 10.5	0.650
Diagnostic catheterization		224 (49.78)	234 (52.00)	0.635
Interventional catheterization	STEMI	48 (10.67)	56 (12.44)	0.537
	NSTEMI-ACS	178 (39.56)	160 (35.56)	0.288
Medical treatment	APT	440 (97.78)	439 (97.56)	0.788
	Anticoagulation	55 (12.22)	52 (11.56)	0.808

^*∗*^Reported as *n* (%), unless indicated otherwise. ACS, acute coronary syndrome; APT, antiplatelet therapy; eGFR, estimated glomerular filtration rate; NSTEMI, non-ST-elevation myocardial infarction.

**Table 2:** Procedural and postprocedural characteristics of the dTRA and TRA groups^*∗*^.

	dTRA	TRA	*P*
Success rate, *n* (%)	432 (96.00)	435 (96.67)	0.814
Access time, min ^*∗∗*^	3.90 ± 2.50	3.10 ± 2.40	0.642
Hemostatic band removal time, min	150.50 ± 50.50	210.60 ± 60.50	0.032
Access site minor bleeding	11 (2.44)	29 (6.44)	0.038
Hematoma	11 (2.44)	8 (1.78)	0.602
Cost of hemostatic band, USD	0.1	59.4	<0.00
Radial artery occlusion after procedure	7 (1.56)	17 (3.78%)	0.033

^*∗*^*n* = 450 in each group; data reported as *n* (%) unless indicated otherwise.  ^*∗∗*^Access time was the time from the subcutaneous local anesthetic to the administration of heparin.

**Table 3:** Characteristics of successful and failed cannulation in patients receiving dTRA ^*∗*^.

		Successful	Failed	*P*
Subjects, *n*		432	18	
Age, years		50.93 ± 9.60	52.34 ± 10.64	0.627
Female		229 (50.89)	11 (61.11)	0.476
Body mass index		24.15 ± 3.57	22.08 ± 3.31	0.034
SBP at the cath lab, mmHg		130.17 ± 13.75	129.71 ± 14.83	0.904
DBP at the cath lab, mmHg		76.14 ± 3.16	74.32 ± 11.22	0.096
Heart rate at the cath lab, bpm		76.28 ± 12.21	76.57 ± 9.44	0.930
Hypertension		60 (13.89)	2 (11.11)	0.540
Diabetes mellitus		34 (7.87)	1 (5.56)	0.682
Current smoking		196 (27.31)	5 (27.78)	0.845
Previous cardiac catheterization		32 (7.41)	2 (11.11)	0.382
LVEF, %		55.4 ± 11.4	56.1 ± 10.3	0.550
Interventional catheterization	STEMI	41 (9.50)	2 (11.11)	0.345
	NSTEMI-ACS	174 (40.28)	7 (38.87)	0.948

^*∗*^Reported as *n* (%), unless noted otherwise. ACS, acute coronary syndrome; APT, antiplatelet therapy; NSTEMI, non-ST-elevation myocardial infraction.

## Data Availability

The data used to support the findings of this study are available from the corresponding author upon request.
